# A Dyadic Approach to Understanding Associations Between Job Stress, Marital Quality, and Dyadic Coping for Dual-Career Couples in Iran

**DOI:** 10.3389/fpsyg.2019.00487

**Published:** 2019-04-18

**Authors:** Reza Fallahchai, Maryam Fallahi, Ashley K. Randall

**Affiliations:** ^1^Department of Psychology, University of Hormozgan, Bandar Abbas, Iran; ^2^Counseling and Counseling Psychology, Arizona State University, Tempe, AZ, United States

**Keywords:** job stress, Iranian dual-career couples, dyadic coping, marital quality, work-family conflict

## Abstract

In Iran, dual-career couples face many stressors due to their demands of balancing work and family. Moreover, the experience of this stress can negatively affect partners’ martial quality. Recent studies have shown the positive impact of dyadic coping on well-being; however, a majority of this research has been conducted with Western cultures. As such, there is a dearth of literature on understanding how supportive and common dyadic coping may have a positive association with work-family stress for couples in Iran. Using a sample of 206 heterosexual dual-career couples from Iran, this study examines the associations between job stress and marital quality, and possible moderating effects of common and perceived partner supportive dyadic coping. As predicted, job stress was negatively associated with marital quality, and this association with further moderated by gender, such that women who experienced greater job stress also reported lower marital quality. Additionally, dyadic coping moderated the association between job stress and marital quality. Common dyadic coping attenuated the negative association between job stress and marital quality. The findings shed light on the possible beneficial effects of teaching supportive and common dyadic coping techniques to dual-career couples in Iran.

## Introduction

Iran is in transition from a society that once focused on agricultural economics to one that is now focused on industrial economy, urbanization, mass media development, and public education (Askari-Nodoushan et al., 2009). In recent decades, family values, structures, and norms have undergone wide-ranging changes due to the shifts in the structure of the Irian society, because of industrialization, urbanization and the expansion of mass media, as well as cultural and value changes, individualism from the dissemination of Western ideas and values (Azadarmaki et al., 2012). These changes have led to shifts in the structure of societies, which can be best observed in changes in the cultural ideals of individualism (Askari-Nodoushan et al., 2009). Examples of this change include the increased age of marriage in 2016 (women: from 18.4 to 23.4; men: from 25 to 27.4), decreasing fertility from 6.3 in 1986 to 1.75 in 2016 (Shojaei and Yazdkhasti, 2017), and increased divorce rates from 8.6 in 1991 to 34.1 in 2018 (Statistics Center of Iran, 2018).

Changes in the Iranian society have also had an impact on the formation and expansion of a nuclear family (Abbasi-Shavazi and McDonald, 2008), wherein husbands were once thought to have the authority in the household due to their economic responsibility, and wives were thought to be responsible for child-rearing (Richter et al., 2014). Due to the modernization of society, women’s increase in educational attainment and rates of employment (Saraie and Tajdari, 2011), women are now thought to have an equal role in all the decision-making of issues related to family (Askari-Nodoushan et al., 2009). The participation of women in the workforce has caused fundamental changes in family and occupational structures, including the increase in dual-career couples (Schaer et al., 2008), so that it has gradually become the dominant model of marital life in most countries (Haddock et al., 2006).

Despite the increase of women in the workforce, which comes along with managing the demands of work-related stress, women in Iran are still expected to attend to their family roles as wives and mothers (Rafatjah, 2011). Consequently, in dual-career families, both partners must perform multiple tasks as well as maintain efforts to create a balance between these roles (Atta et al., 2013). Research on 155 dual-career couples in Bangladesh has shown that childcare, work-family conflict, family-work conflict, and marital relations are the most important challenges for dual-career couples (Sultana et al., 2014). Consequently, the balance of work and family roles can be stressful (Rafatjah, 2011), and may lead to conflicts between partners (Soleimanian and Nazari, 2007; Nazari and Goli, 2008; Oreizi et al., 2011), which over time can lead to decreased marital quality.

Given the overwhelming number of dual-career couples in Iran (Khosravi et al., 2010; Motahari et al., 2012; Fallahchai and Khaluee, 2016; Mazhari et al., 2016), investigating the unique stressors these couples may face is an important concern for mental health practitioners working with these couples (Saginak and Saginak, 2005). Few studies have investigated marital quality in dual-career couples in a collectivist context (Quek et al., 2011), which leaves a dearth of understanding on factors can affect partners’ marital quality. Given the changing cultural climate in Iran, it is necessary for relational scholars to examine ways in which dual-career couples can cope with stress in order to possible reduce marital dissatisfaction (Soleimanian and Nazari, 2007). Additionally, dyadic coping has been found to moderate the associations between work-family conflicts in Canada (Lapierre and Allen, 2006), as well as preventing the harmful effects of stress on relational functioning and physical and mental health (Levesque et al., 2014; Merz et al., 2014).

### Associations Between Job Stress and Marital Quality

Job stress is defined as a reaction to the experience of stressors related to work domains (Wierda-Boer et al., 2009), which can be accompanied by role-overload due to occupational and family responsibilities. Not surprisingly, job stress can also affect within the family due to stress spillover and crossover (Neff and Karney, 2005), ultimately leading to a decrease in marital quality in both partners. Stress spillover refers to how the stress experienced from an aspect of life (e.g., occupation) spills over causing stress to another aspect (e.g., family) (Geurts and Demerouti, 2003). For example, when a person has a stressful day at work this may affect the way they interact with their partner (e.g., shutting down), causing stress at home. Work-family spillover is defined as the transfer of the effects of work and family on one another that generate similarity between work and family (Edwards and Rothbard, 2000) and work-family spillover transfers from one domain (e.g., occupation) to another domain (family) (Haines et al., 2006; Schaer et al., 2008). Stress spillover in marital relationships can lead to negative behaviors, such as anger toward the partners (Schulz et al., 2004), which, can negatively affects marital satisfaction (Randall and Bodenmann, 2017). Stress crossover refers to the interpersonal transfer of stress from one partner to another (Haines et al., 2006). For example, one partner’s experience of stress can affect their partner’s experience as well (Randall et al., 2017). Bodenmann et al. (2007) demonstrated that stress outside the relationship (external stress; Randall and Bodenmann, 2009) significantly triggers stress within the relationship (internal stress), which is commonly found to be associated with marital quality.

Not surprisingly, the experience of job stress has been found to reduce marital quality in both partners (Obradovic and Cudina-Obradovic, 2009). A majority of research in this domain has been conducted in the United States or with Western samples and has found that men were affected by work-family conflict as much as women, however, women were more likely to be affected by family-work conflict than men (e.g., Tatman et al., 2006). However, recent research is starting to examine these associations with non-Western samples. For example, Sandberg et al. (2012) examined the association between family-to-work spillover job satisfaction and health using a sample of 1026 married workers in Singapore. Results of this study showed that marital distress was a significant predictor of job satisfaction and health. Taken together, given the negative associations between job-stress and marital quality (Neff and Karney, 2007; van Steenbergen et al., 2011) and increased rates of divorce in Iran (National Organization for Civil Registration, 2017), it is important to consider ways in which couples could cope with stress that may prevent the harmful effects of stress on relational well-being (Randall and Bodenmann, 2009, 2017; Merz et al., 2014).

### Moderating Associations of Dyadic Coping

The conceptualization of stress as a dyadic construct, one that affects both partners in a romantic relationship (Randall and Bodenmann, 2009). Given this conceptualization, partners can attempt to cope with stress by engaging in (positive) dyadic coping. Specifically, dyadic coping refers to the ways in which partners cope with stress in the context of their relationship (Bodenmann et al., 2011). Although positive and negative forms of dyadic coping exist (see Bodenmann, 2005), here we focus specifically on positive forms of dyadic coping given its strong association with relational well-being for couples around the world (Falconier et al., 2016). Additionally, a recent meta-analysis by Falconier et al. (2015) found that supportive and common dyadic coping were found to be powerful (positive) predictors of relationship satisfaction (e.g., Bodenmann and Cina, 2006; Ruffieux et al., 2014).

Positive forms of dyadic coping can be classified into three categories: *supportive dyadic coping, delegated dyadic coping*, and *common dyadic coping* (Bodenmann, 2005). *Supportive dyadic coping* refers to the efforts that one couple makes to express empathic understanding, solidarity with his/her partner, and providing practical. For example, if a partner is under stress, his/her partner may respond by expressing empathy and then providing practical advice on how to help cope with the stress. *Delegated dyadic coping* refers to a new division of tasks in which one partner asks for practical support. For example, a partner takes over certain tasks of the partner when his/her partner asks for help. Lastly, *common dyadic coping* represents the joint efforts of couples to deal with stress. For example, both partners engage in joint problem solving when they face a stressful situation (e.g., work-family conflict). Furthermore, research suggests that common dyadic coping plays an important role in reducing negative daily stress (e.g., Bodenmann et al., 2011; Falconier et al., 2015), increasing the quality of the relationship, reducing symptoms of depression and distress in both couples (Rottmann et al., 2015).

Prior research has focused on the direct association between dyadic coping and marital quality (e.g., Iafrate et al., 2012; Falconier et al., 2015; Gasbarrini et al., 2015), as well as on the indirect association (i.e., moderation) between variables (e.g., Falconier et al., 2013; Levesque et al., 2014; Herzberg and Sierau, 2016).

#### Direct Associations

Bodenmann et al. (2006a) who examined the association between dyadic coping and marital quality among 90 Swiss couples over a 2 year period found that dyadic coping was positively correlated with relationship quality for couples. Additionally, using a sample of 187 heterosexual couples from Switzerland, Levesque et al. (2014) investigated dyadic empathy, dyadic coping, and relationship satisfaction. Results from this study showed that, among men, perspective-taking significantly increased their partner’s desire to use positive dyadic coping strategies. For female, empathy increased their partners’ coping strategies.

#### Indirect Associations

Dyadic coping has also been shown to have moderating effects on the association between stress and individual and relational well-being. For example, supportive and common dyadic coping were found to reduce the negative associations between immigration stress on relationship satisfaction for 104 Latino immigrant couples in the United States, especially for women (Falconier et al., 2013). The results of the study by Merz et al. (2014), examined the moderation role of dyadic coping in association between internal stress and relationship satisfaction on 131 couples, showed that dyadic coping reduced the effects of chronic stress on relationship satisfaction especially in women. Most recently, Hilpert et al. (2018) studied stress and coping processes at both between- and within-person levels in 84 dual-earning couples in China. The results of this study indicated that at the between persons level, both in men and women, the association between stress and relationship outcomes was decreased if the partner provided more support, but at the level of within persons, the results indicated that partner support had only a significant buffer effect in women. Taken together, supportive dyadic coping has been shown to be effective in reducing stress and improving the quality of relationships (Vedes et al., 2013); however, this has yet to be examined in dual- career couples, especially those from Iran, which is the goal of the present study.

## The Present Study

The goal of the present study is to investigate the association between job stress and marital quality in dual-career couples from Iran. Additionally, given the robust positive associations between dyadic coping and relational outcomes found across cultures (Falconier et al., 2016), we also examine how supportive and common dyadic coping may moderate the association between job stress and marital quality. To do so, we collected dyadic data from both partners in a romantic relationship, which allows us to examine both actor and partner effects (Kenny et al., 2006). Actor effects refer to the associations of partner’s reports of their independent variable (job stress) on their dependent variable (marital quality), whereas partner effects refer to the associations between how one partner’s reports job stress are associated with their partner’s marital quality.

In sum, we tested the following hypotheses (H):

H1: In line with research that has found a positive association between job stress and marital conflict in dual-career couples from the United States and Western Europe (Michel et al., 2009; Allen and Finkelstein, 2014; Fellows et al., 2016; Yucel, 2017), it is hypothesized that a partner’s job stress will be negatively associated with one’s own (actor effect) and their partner’s marital quality (partner effect).

H2a: Based upon prior studies suggesting that supportive dyadic coping moderates the negative association between stress and relationship quality (Bodenmann, 2005; Bodenmann et al., 2010; Vedes et al., 2013; Rottmann et al., 2015; Breitenstein et al., 2018) it is hypothesized that perception of partner’s of supportive dyadic coping will moderate the association between job stress and his/her own marital quality. Moreover, it is hypothesized that actual reports of supportive dyadic coping will be associated with partner’s reports of marital quality (partner effect).

H2b: Based on prior studies that have found a positive association between common dyadic coping and relationship quality (Bodenmann, 2005; Bodenmann et al., 2010; Papp and Witt, 2010), it is hypothesized that common dyadic coping will moderate the association between job stress and marital quality, such that for individuals who perceive their partner as engaging in common dyadic coping, they will also show a positive association between their coping with job stress and their marital quality (actor effects). Additionally, we hypothesize that we will also find a positive association between self-reported job stress and perception of marital quality.

### Gender Differences

Although studies have found men and women report the same levels of stress in work-family conflict (Barnett and Gareis, 2006; Martinengo et al., 2010), men and women show different behavioral patterns in response to stress; women showed a higher level of negative spillover than men (Mennino et al., 2005). Related to job-stress in particular, Barling et al. (2004) have shown that there are important gender differences in the degree to which job and family stress is transmitted to negative family processes, including cognitions, behaviors, and interactions within the family that lead to negative outcomes. Given this, we also examine whether gender will moderate the association between job stress and marital quality.

## Materials and Methods

### Participants and Procedure

This research was reviewed and approved by the ethics and research committee of Hormozgan University prior to the start of data collection. All participants provided written and informed consent. They were recruited in person from civil institutions, local police, education and social services in Shiraz, Iran. Participants had to meet the following criteria in order to participate (a) married for at least 2 years, (b) both of the partners had to have been working at least 2 years, and (c) have full-time employment status. Eligible couples were given two packages of research questionnaire in separate envelopes with a unique ID. Participants were asked to fill in their questionnaires independently from their spouse and send back the questionnaires upon completion.

Data were collected from 238 couples; however, 32 couples were removed from the current analysis for having incomplete data. The final sample consisted of 206 heterosexual couples (*n* = 412 individuals). On average, the men were 35.7 years old (*SD* = 9.1 years; range: 25–62 years) and women were 31.1 years old (*SD* = 9.3 years; range: 21–51 years). The sample was highly education with 62.6% of participants reporting having B.A. degrees, 26.2% had M.A. and Ph.D. degrees, and 11.2% had high school diplomas. Participants reported being married for an average of 11 years (*SD* = 7.2). The average number of children was 2 ranging from 1 to 3.

### Measures

#### Demographic Information

Standard demographic information relating to age, gender, level of education, length of relationship, number of children was collected. See Table 1 for descriptive information.

**Table 1:** Descriptive of study variables.

Variable	Husbands (*N* = 203)			Husbands (*N* = 203)			Paired *t*	*p*	Cohen’s d
				
	M	SD	Range	M	SD	Range			
Age	32.19	4.19	26–41	27.42	3.76	23–39	0.56	0.60	0.02
Relationship duration	11.60	9.10	2–23	12.10	10.20	3–25	0.51	0.58	0.02
Number of children	2.00	1.10	1–3	2.20	1.20	1–4	0.49	0.71	0.012
Job stress	90.00	12.45	35–175	86.07	11.03	35–175	-2.04*	0.03	0.31
Marital quality	117.16	17.24	0–151	109.00	13.70	0–151	1.90*	0.02	0.28
Perceived partner supportive dyadic coping	19.46	3.58	5–25	19.23	2.94	10–50	1.16	0.21	0.08
Common dyadic coping	19.01	3.22	5–25	19.55	3.23	5–25	1.30	0.38	0.15


*^∗^*p* < 0.05.*

#### Job Stress

Participant’s perception of job stress was measured using the Persian version of the Health and Safety (HSE) Management Standards Indicator Tool (HSE-MS IT; Cousins et al., 2004). The HSE-MS IT is a 35-item on a 5-point Likert type scale (1 = *never* to 5 = *always*) developed to measure work-related stress risk factors at an organizational level (Marcatto et al., 2014). This measure showed good reliability in the current study for men and women (α = 0.82 and 0.85, respectively).

#### Marital Quality

Participants’ reports of marital quality were measured using the Persian version of Dyadic Adjustment Scale (Sanai Zakir, 2000; DAS; Spanier, 2001). The DAS includes 32 items used to assess partners’ marital quality (e.g., Fişiloǧlu and Demir, 2000; Chiara et al., 2014; Bachem et al., 2018). Twenty-seven items are rated on a 6- point Likert scale (15 items: 0 = *always disagree* to 5 = *always agree*; 12 items 0 = *never* to 5 = *all the time*); two items are on a 5-point Likert scale (0 = *never* to 4 = *everyday*); two items are yes/no type questions (0 = *no* 1 = *yes*); and one is on a 7-point Likert scale (0 = *extremely unhappy* to 7 = *perfect*). Items are summed, wherein higher scores are reflective of greater marital quality. Results of current study showed good reliability for men and women (α = 0.86 and 0.87, respectively).

#### Dyadic Coping

The Persian version of the Dyadic Coping Inventory (DCI; Fallahchai et al., 2017) was used to measure participant’s reports of dyadic coping. The DCI is a self-report instrument consisting of 37 items, with responses arranged on a 5-point likert-type scale (*1 = never to 5 = always*). The DCI contains six subscales to measure each partner’s stress communication and specific dyadic coping; however, for the purpose of our study, we examined the following: emotion-focused dyadic coping, problem-focused dyadic coping, and common dyadic coping. To create a composite score of *perceived partner supportive dyadic coping*, we took the average of emotion-focused and problem-focused supportive dyadic coping. For each area assessed, participants reported on their own and their perceived partner behaviors; reports of perceived partner dyadic coping were used in the present analysis. This measure showed good reliability for *perceived partner supportive dyadic coping* for men and women (α = 0.83 and 0.84, respectively), and *common dyadic coping* for men and women (α = 0.88 and 0.89, respectively).

#### Control Variables

Age (e.g., Michel et al., 2009; Spell et al., 2009), relationship length, and the number of children (e.g., Hassan et al., 2010; Wang et al., 2010; Mache et al., 2015) have been previously found to be negatively associated with work-family conflicts. As such, we controlled for these variables in our analysis.

### Statistical Analyses

Dyadic data – data collected from two partners – contains sources of interdependence between partners’ reports (Kenny et al., 2006). Analyses were run with Actor-Partner Interdependence-Model (APIMs) (Cook and Kenny, 2005) which allows researchers to control for the interdependence between reporting partners’ scores, and also examine both actor and partner effects. To analyze both actor and partner effects, we used Structural Equation Modeling (SEM) for distinguishable dyads (e.g., men and women) because SEM allows for the estimation the association between variables free from measurement error, while also including the examination of the goodness of fit of the base models and the measurement structure of all study variables simultaneously (Ledermann and Kenny, 2017).

In order to evaluate model fit, we used the Root Mean Square Error of Approximation (RMSEA; 0.01 = excellent fit; 0.05 = good fit; 0.08 = mediocre fit; MacCallum et al., 1996), and Comparative Fit Index (CFI; 0.95 = excellent fit; 0.90 = adequate fit; Hu and Bentler, 1999). Each model contained the control variables noted above. All analyses were conducted using AMOS 21 (Arbuckle, 2006).

## Results

### Descriptive Statistics

Descriptive statistics for the study variables are presented in Table 1. Results showed significant gender differences in self-reported job stress; wives reported significantly higher scores in job stress (*t* = -2.04, *p* = 0.03). Interestingly, compared to wives, husbands reported higher marital quality (*t* = 1.90, *p* = 0.02). We did not find differences between husbands and wives reports of engaging in partner supportive dyadic coping.

Significant correlations among the scales ranged from (-0.43 < *r* > 0.77) for both husbands and wives. Table 2 shows correlations among measured variables for husbands (above the diagonal), for wives (below the diagonal).

**Table 2:** Correlations between study variables.

	1	2	3	4	5	6	7
1. Age	0.15	0.08	0.09	-0.18	0.09	0.14	0.07
2. RD	0.04	1.00	-0.13	0.14	-0.10	-0.11	-0.14
3. NC	0.08	-0.13	1.00	0.07	-0.21^*^	-0.17	-0.15
4. JS	0.08	0.12	0.17	0.79^**^	-0.42^**^	-0.41^**^	-0.36^**^
5. MQ	0.08	0.08	0.10	-0.34^**^	0.77^**^	0.75^**^	0.62^**^
6. PSDC	0.10	-0.12	0.90	-0.42^**^	0.70^**^	0.78^**^	0.67^**^
7. CDC	0.11	-0.14	-0.15	-0.30^**^	0.63^**^	0.72^**^	0.74^**^


*RD, Relationship Duration; *NC*, Number of children, *JS*, Job stress, *MQ*, Marital Quality, *SDC*, Perceived Partner Supportive Dyadic Coping, *CDC*, Common Dyadic Coping. Husbands’ correlations are presented above the diagonal and wives’ correlations are presented below the diagonal. Between-partner correlations are presented across the diagonal. ^∗∗^, 0.01, ^∗^, 0.05.*

### Associations Between Job Stress and Marital Quality

A model with the direct actor and partner effects of job stress predicting change in spouses’ marital quality was examined. Gender was included in the models to test whether the associations between job stress and marital quality differed between husbands and wives. The model fit well: *χ*^2^ = 8.789, *p* < 0.45, CFI = 0.95, RMSEA = 0.02.

**FIGURE 1:**
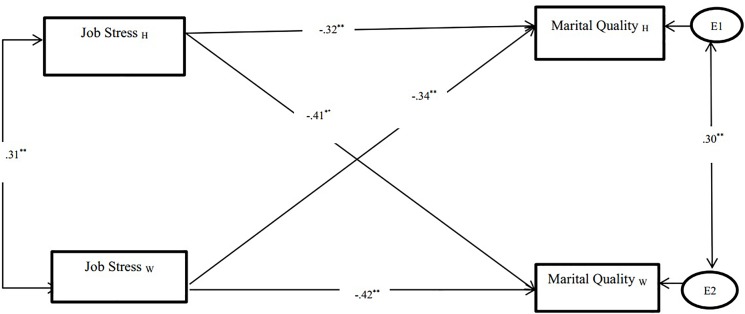
Association between job stress and marital quality. ^∗∗^*p* < 0.01; W, Wives; H, Husbands.

It was hypothesized that job stress would have a main effect on marital quality after controlling for age, marital duration, and number of child. Results indicated that there was a significant negative association between one’s own job stress and marital quality for both husbands and wives (actor effect; husbands: β = -0.32, *p* < 0.001; wives: β = -0.42, *p* < 0.001). Additionally, we found partner effects for job stress and marital quality for both husbands (β = -0.41, *p* < 0.001) and wives (β = -0.34, *p* < 0.001); one’s reports of job stress was negatively associated with their partner’s reports of marital quality (see Figure 1).

Moreover, results indicated the actor association differed by gender. For example, significant gender differences were found in associations between job stress and marital quality (β = -0.39, *p* < 0.01), for wives. Specifically, when wives reported greater job stress they also reported lower marital quality. However, gender did not moderate the partner effect.

### Moderating Associations of Dyadic Coping

#### Perceived Partner Supportive Dyadic Coping (H2a)

The Goodness-of-fit for the model with perceived partner supportive dyadic coping as the moderator was very good: *χ*^2^ = 6.45; *p* = 0.451; with CFI = 0.92 and RMSEA = 0.04. All the actor effects were significant and in the expected direction, but the partner effects were not significant.

##### Actor Effects

The structural path from the interaction between husbands’ perceived partner supportive dyadic coping and husbands’ job stress to husbands’ marital quality was significant (β = -0.44, *p* < 0.001), which suggests that when husbands perceive their wife as engaging in supportive dyadic coping they report greater marital quality. Additionally, results found that perceived partner supportive dyadic coping moderated the association between job stress and marital quality, this effect was significant for wives (β = -0.47, *p* < 0.001).

##### Partner Effects

**FIGURE 2:**
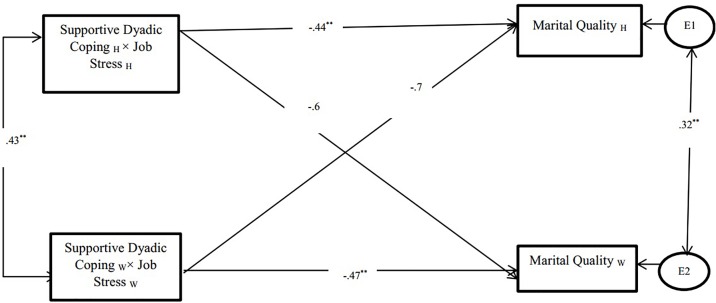
Association between perceived partner supportive dyadic coping and marital quality. ^∗∗^*p* < 0.01; W, Wives; H, Husbands. “Supportive dyadic coping” was measured by partner’s perception of their partner’s engagement in dyadic coping.

Results showed that the interaction between husbands’ perceived partner supportive dyadic coping and husbands’ job stress to wives’ marital quality was not significant (β = -0.6, *p* > 0.05), and the interaction between wives’ perceived partner supportive dyadic coping and wives’ job stress to husbands’ marital quality was not significant (β = -0.7, *p* > 0.05). Therefore, the partner effects were not significant both for husbands and wives (see Figure 2).

##### Common Dyadic Coping (H2b)

Results of estimating the APIM revealed very good Goodness-of-fit for the model (*χ*^2^ = 5.23, *p* = 0.32 with CFI = 0.96 and RMSEA = 0.031).

##### Actor Effects

The structural path from the interaction between common dyadic coping and job stress to marital quality was significant. This effect was found for both husbands (β = -0.44, *p* < 0.001) and wives (β = -0.47, *p* < 0.001).

##### Partner Effects

**FIGURE 3:**
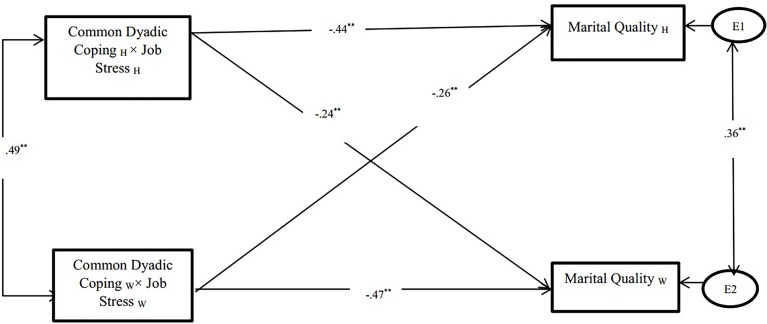
Association between common dyadic coping and marital quality. ^∗∗^*p* < 0.01; W, Wives; H, Husbands.

Results showed that the interaction between husbands’ common dyadic coping and husbands’ job stress to wives’ marital quality was significant (β = -0.24, *p* < 0.05). Moreover, the interaction between wives’ common dyadic coping and wives’ job stress to husbands’ marital quality was significant (β = -0.26, *p* < 0.05) (see Figure 3).

Taken together, results revealed that perceived partner supportive dyadic coping and common dyadic coping moderated the negative association between job stress and marital quality in expected directions.

## Discussion

Given the change of social-cultural structure in Iran (Askari-Nodoushan et al., 2009; Azadarmaki et al., 2012), and the increase of dual-career couples (Soleimanian and Nazari, 2007; Ghodrati, 2015), the aim of this study was to investigate the association between job stress and marital quality for dual- career couples, and assess possible moderating associations of supportive and common dyadic coping. Results from this study largely support our hypotheses, however, interesting gender differences emerged, which are explained below.

### Associations Between Job Stress and Marital Quality

We hypothesized that a partner’s job stress would be negatively associated with one’s own marital quality (actor effect) and their partner’s marital quality (partner effect). Findings of this study found that both wives and husbands in a dual- career marriage report similar levels of job stress, and these reports were similarly associated with marital quality (both actor and partner effects). This finding is in line with the results of previous studies (Anafarta, 2011; Šimunic and Gregov, 2012; Efeoǧlu and Ozcan, 2013) suggesting dual- career couples experience a lot of job stress, which is associated with marital quality (Buck and Neff, 2012; Sandberg et al., 2012).

### Moderating Effects of Perceived Dyadic Coping

We hypothesized that supportive and common dyadic coping would moderate the association between job stress and their own marital quality (actor effect) and partner’s reports of marital quality (partner effect). Below we expand upon the results from these models.

#### Perceived Partner Supportive Dyadic Coping

Data from this study supported our hypothesis, suggesting that perceived partner supportive dyadic coping moderated the negative association of job stress and marital quality for husbands and wives. Said differently, when individuals reported greater partner’s supportive dyadic coping, they also experienced higher level of marital quality. These results are consistent with previous studies (e.g., Wunderer and Schneewind, 2008; Papp and Witt, 2010; Falconier et al., 2013; Herzberg, 2013; Nicholls and Perry, 2016), which have found supportive dyadic coping to have a beneficial effect on marital quality (Bodenmann et al., 2006b, 2016; Falconier et al., 2013; Breitenstein et al., 2018).

#### Common Dyadic Coping

Data from this study supported our hypothesis, suggesting that common dyadic coping moderated the negative association of job stress (actor and partner effect) for both husbands and wives. These results are in line with previous studies that have found that common dyadic coping may play a moderating role in association between stress and marital outcomes (i.e., relationship satisfaction, marital quality) (e.g., Bodenmann, 2005; Bodenmann et al., 2010; Falconier et al., 2013). Given that common dyadic coping refer to partners’ perception of handling stressful situations, these results support understanding stress and coping as a dyadic context (Randall and Bodenmann, 2009, 2017). In situations where one of the partners, or both, faces a significant stressor, viewing stress as a dyadic stress (i.e., “our stress”) and engaging in common dyadic coping can help partners cope with the stress, by fostering a sense of “we-ness” within the couple (Vedes et al., unpublished). Therefore, this strong association between positive dyadic coping techniques and ability to cope with stress suggests that the way in which couples manage and interact with stress and conflict in their marital life is considered as the most important determinants of marital satisfaction (Vedes et al., 2013), and the satisfaction of the relationship may dependent on positive dyadic coping during times of distress (Falconier et al., 2015).

### Role of Gender

Another goal of the present study was to examine possible gender differences between job stress and marital quality. Results from this study revealed that when wives reported greater job stress they also reported lower marital quality, however, this effect was not found for husbands. One’s is a very important culture is a very important factor for predicting gender differences in the coping process between couples (Hilpert et al., 2016). In Iran Khojasteh mehr et al. (2013) in their research with 150 couples, found that dyadic coping in women had a greater effect on their marital satisfaction than men, because the support that women under stress receive from their husbands has a great influence on the quality of their marital life.

Additionally, women may engage in greater dyadic coping behaviors due to their greater attentiveness to their partner’s needs (Bodenmann et al., 2006a), as women are thought to be more sensitive to changes in their marital relationships (Bodenmann et al., 2004). This greater engagement in dyadic coping behaviors may be particularly true for couples who come from a society wherein men and women carry different roles and responsibilities in the relationship (see Hilpert et al., 2016).

### Limitations and Future Directions

Although this study is one of the few studies that has used a dyadic sample of dual-career couples from Iran to examine associations between job stress and marital quality, it is notwithstanding limitations. First, data for this study was based on cross-sectional data, which limits our ability to make causal inferences and further test associations between stress spillover and crossover. To better address for stress spillover (i.e., external stress to internal stress; see Randall and Bodenmann, 2009) and crossover future research should utilize longitudinal data (e.g., Bodenmann and Cina, 2006; Bodenmann et al., 2006a). Second, this study relied on the use of self-report assessments, which may contain bias (Spector, 1994). As such, future research is encouraged to use a multi-method approach that includes more objective measures, such as observational measures and interview methods, which may provide a better understanding of the nature of how and when supportive dyadic coping is utilized especially given the cultural context. Third, it is important to examine other variables that may further moderate the association between job stress and marital quality. One such variable is the presence of children in the home. Having a child affects the work-family conflict (Mennino et al., 2005), and negatively affects the individual’s job performance (Patel et al., 2006), as such the presence of children may. Lastly, this study chose to focus on supportive and common dyadic coping due to its robust positive associations with relationship well-being (see Falconier et al., 2015). To further understand the role of dyadic coping in the context of dual-career couples, future research should also examine other types of dyadic coping (e.g., delegated and negative dyadic coping), which may help relationship researchers and clinicians working with couples identify other forms of effective coping on the relationship between job stress and marital quality. Also, considering the cultural differences between Iran and Western countries regarding gender roles and its possible effects on family-work conflict of the couples, it is suggested that future research would measure specific gender roles.

## Conclusion

The number of dual- career couples is increasing in Iran (Ghodrati, 2015). In addition to stress common to all couples (Jackson et al., 2016), some couples may experience higher levels of stress due to work-family conflicts (Nohe et al., 2015), which can have negative implications on their relational well-being (Randall and Bodenmann, 2009, 2017). Recent research cross-culturally has shown that supportive and common dyadic coping have buffering effect on reducing the impact of stress and can enhance marital quality (Bodenmann et al., 2010; Falconier et al., 2016).

Perhaps not surprisingly, the results of this study found that job stress was negatively associated with marital quality; however, perceived partner supportive and common dyadic coping moderated the association. The findings of this study have improved our understanding of stress processes in marital quality of dual- career couples. The findings of this study improve our understanding of stress and coping processes for dual-career couples in Iran, and the importance of engaging in supportive and common dyadic coping. These findings suggest that dyadic coping plays a very important role both in reducing stress and improving the quality of relationships in dual-career couples in Iran. The findings of this study are important implications for relationship researchers and clinical experts in understanding the effects of work-family stress on marital quality in dual-career couples and their gender differences in their rate and effect of job stress. Moreover, teaching coping skills can be effective both in reducing stress and improving the quality.

## Author Contributions

MF organized the database and performed the statistical analysis. RF wrote the first draft of the manuscript and performed the statistical analysis. MF, RF, and AR wrote the sections of the manuscript. All authors contributed to the manuscript revision, read, and approved the submitted version.

## Conflict of Interest Statement

The authors declare that the research was conducted in the absence of any commercial or financial relationships that could be construed as a potential conflict of interest.
